# Flow cytometry-based targeted diagnostics for rapid assessment of daunorubicin resistance in acute myeloid leukemia

**DOI:** 10.1038/s41598-025-30844-2

**Published:** 2025-12-09

**Authors:** Aleksandra Kaczorowska, Przemysław Sareło, Marlena Gąsior-Głogowska, Ewa Zioło, Paweł Karpiński, Łukasz Łaczmański, Marta Sobas, Tomasz Wróbel, Halina Podbielska, Marta Kopaczyńska, Wojciech Kałas

**Affiliations:** 1 Department of Biomedical Engineering, Faculty of Fundamental Problems of Technology, Wrocław University of Science and Technology, Wybrzeże Wyspianskiego 27, 50-370 Wrocław, Poland; 2 Pre-clinical Research Center, Wrocław Medical University, Karola Marcinkowskiego 1, 50- 368 Wrocław, Poland; 3 Department of Experimental Oncology, Ludwik Hirszfeld Institute of Immunology and Experimental Therapy, Polish Academy of Sciences, Rudolfa Weigla 12, 53-114 Wrocław, Poland; 4 Department of Genetics, Wrocław Medical University, Karola Marcinkowskiego 1, 50-368 Wrocław, Poland; 5 Laboratory of Genomics and Bioinformatics, Ludwik Hirszfeld Institute of Immunology and Experimental Therapy, Polish Academy of Sciences, Rudolfa Weigla 12, 53-114 Wrocław, Poland; 6 Department and Clinic of Hematology, Cellular Therapies and Internal Medicine, Faculty of Medicine, Wrocław Medical University, Wybrzeże Pasteura 4, 50-367 Wrocław, Poland

**Keywords:** Acute myeloid leukemia, Daunorubicin resistance, Flow cytometry assay, Ex vivo culture, Drug response testing, Precision medicine, Biomarkers, Cancer, Computational biology and bioinformatics, Drug discovery, Oncology

## Abstract

Acute myeloid leukemia (AML) is an aggressive hematologic malignancy where chemoresistance, particularly to daunorubicin (DNR), frequently leads to treatment failure and relapse. Current resistance prediction methods are often slow and inaccessible in clinical settings, delaying crucial treatment adjustments. There is an urgent need for rapid, reliable, and clinically accessible diagnostic tools to identify DNR resistance early, enabling personalized therapeutic strategies and improving patient outcomes. To address this problem, we developed a rapid flow cytometric assay to detect reduced intracellular DNR accumulation, a marker of drug resistance. This was complemented by FTIR spectroscopy, confocal microscopy, and proteomic profiling to validate findings and explore underlying mechanisms in drug-resistant and sensitive cells. The flow cytometry method demonstrated that the novel Sensitivity Index (S-index) accurately predicts DNR resistance after short drug exposure and correlates strongly with IC_50_ values. Complementary FTIR and confocal microscopy revealed distinct biochemical and structural differences between resistant and sensitive cells. Proteomic analysis suggests that increased drug metabolism contributes to DNR resistance, supporting the flow cytometry data. In summary, our findings demonstrate that flow cytometry provides a reliable and clinically accessible tool for targeted diagnostics, crucial for optimizing patient-specific treatment strategies based on rapid assessment of daunorubicin resistance in AML.

**Supplementary Information:**

The online version contains supplementary material available at 10.1038/s41598-025-30844-2.

## Introduction

Acute myeloid leukemia (AML) is an aggressive neoplasm of hematopoietic stem cells, characterized by the infiltration of the bone marrow by leukemic blasts, leading to the suppression of normal haematopoiesis. The aetiology of AML is heterogeneous and highly complex^1^. Multiple genetic alterations and somatic mutations contribute to the development of AML, with cytogenetic profiles and mutational status; summarized by the European LeukemiaNet (ELN) risk score, having the greatest impact on prognosis^2–4^. Primary resistance and early relapse represent major challenges in the treatment of AML. Primary resistant leukemia accounts for approximately 10–40% of cases at diagnosis, while relapse occurs in 40–50% of younger patients and in the majority of elderly AML patients^5^. Chemotherapy resistance continues to pose a significant challenge in clinical practice^6,7^.

Currently, in clinics there is a lack of cheap, rapid and reliable tools to identify AML patients at high risk of early relapse at the time of diagnosis. The development of such tests would help prevent overtreatment and enable the timely initiation of novel therapeutic strategies. The ex vivo evaluation of chemotherapeutic drug efficacy as a predictor of success in anticancer therapy is a well-established concept with a long-standing history^8–11^. This concept aligns well with the overarching strategy of personalized therapy, which seeks to address challenges posed by cancer heterogeneity^12,13^. Accordingly, numerous studies have demonstrated that in vitro assays can successfully predict the chemoresistance of cancer cells. Blom et al. reported an overall sensitivity of 88% (CI 86–90%) and a specificity of 72% (CI 68–75%) for ex vivo tests, irrespective of the cytotoxicity assessment method used^14^. For example, studies involving over 400 lung cancer patients demonstrated that those with higher in vitro drug sensitivity experienced significantly longer disease-free survival (18 months vs. 8.5 months) compared to patients exhibiting in vitro drug resistance^15^. A multicenter exploratory trial further demonstrated the feasibility of using ex vivo tests to select gastric cancer patients for postoperative adjuvant chemotherapy with 5-fluorouracil, with responders showing significantly better therapeutic outcomes compared to non-responders^16^. Similarly, a study conducted on samples from 125 leukemia patients showed that ex vivo testing can effectively predict primary drug resistance, guide clinical decision-making, and help prevent inappropriate treatment^17^.

Nevertheless, the methodologies commonly employed in these studies primarily assess cell death and include assays such as MTS, colony formation, and sulforhodamine B^8,18–20^. Notably, all these methods involve culturing cells in the presence of the tested drug, requiring highly specialized personnel and equipment. Such experimental setups are typical of research laboratories but not of clinical diagnostic laboratories or hospitals. Consequently, most studies demonstrating the benefits of ex vivo cancer testing were conducted on relatively small patient cohorts and were rarely expanded, as completing even a modest study demands considerable effort and resources^15^. These practical barriers limit the broader application of ex vivo testing in patient care and lead to insufficient data on its efficacy and accuracy.

Anthracyclines, as daunorubicin (DNR), or idarubicin are the most common drugs used in anti-AML therapy^4,21,22^. DNR and other anthracyclines are cytotoxic antimitotics that enter cells via passive diffusion. Their complex mechanism of action includes: (a) forming covalent complexes with DNA, (b) inhibiting topoisomerase II, (c) interfering with RNA and DNA polymerase activity, (d) binding to cellular components beyond DNA, such as the endoplasmic reticulum, (e) generating reactive oxygen species in the presence of cytochrome P450 reductase and NADH dehydrogenase, (f) disrupting proteasome function, and (g) inducing ceramide synthesis via ceramide synthase and serine palmitoyltransferase^23,24^. Accordingly, resistance to anthracyclines is a multifactorial phenomenon involving various mechanisms, including: (a) multidrug resistance (MDR), (b) DNA repair, (c) alterations in topoisomerase II activity, and (d) drug metabolism^25^.

The broad spectrum of activity of anthracyclines is the reason for their excellent clinical efficacy. However, this same complexity makes it challenging to identify resistance to anthracyclines through molecular and cytogenetic analysis.

One of the frequently used and easily accessible methods in clinical environment is flow cytometry. It can be suitable also for determining the drug resistance in AML. There were some attempts reported on use of cytometry for leukemia studies and assessing drug sensitivity^26^. It was shown that flow cytometry may be apply to study of some surface markers expression in a blood of patients^27^. It was also demonstrated that automated flow cytometry allows to evaluate the sensitivity of leukemia cells to multiple chemotherapeutic drugs ex vivo. Although an analysis time of 48–72 h is clinically advantageous compared to methods requiring longer incubation periods, it remains relatively long for making timely, patient-focused clinical decisions^28^.

Therefore, our study focuses on the rapid assessment of leukemic cell chemoresistance to daunorubicin by detecting intracellular drug content using flow cytometry following brief drug exposure. With future clinical application in mind, the goal is to develop procedure that is fast and reliable, requiring minimal or no demanding cell culture steps and no reliance on specialized equipment.

## Results

### Low- IC_50_ and high- IC_50_ cells differ in the acquisition of DNR-related fluorescence

**Fig. 1:**
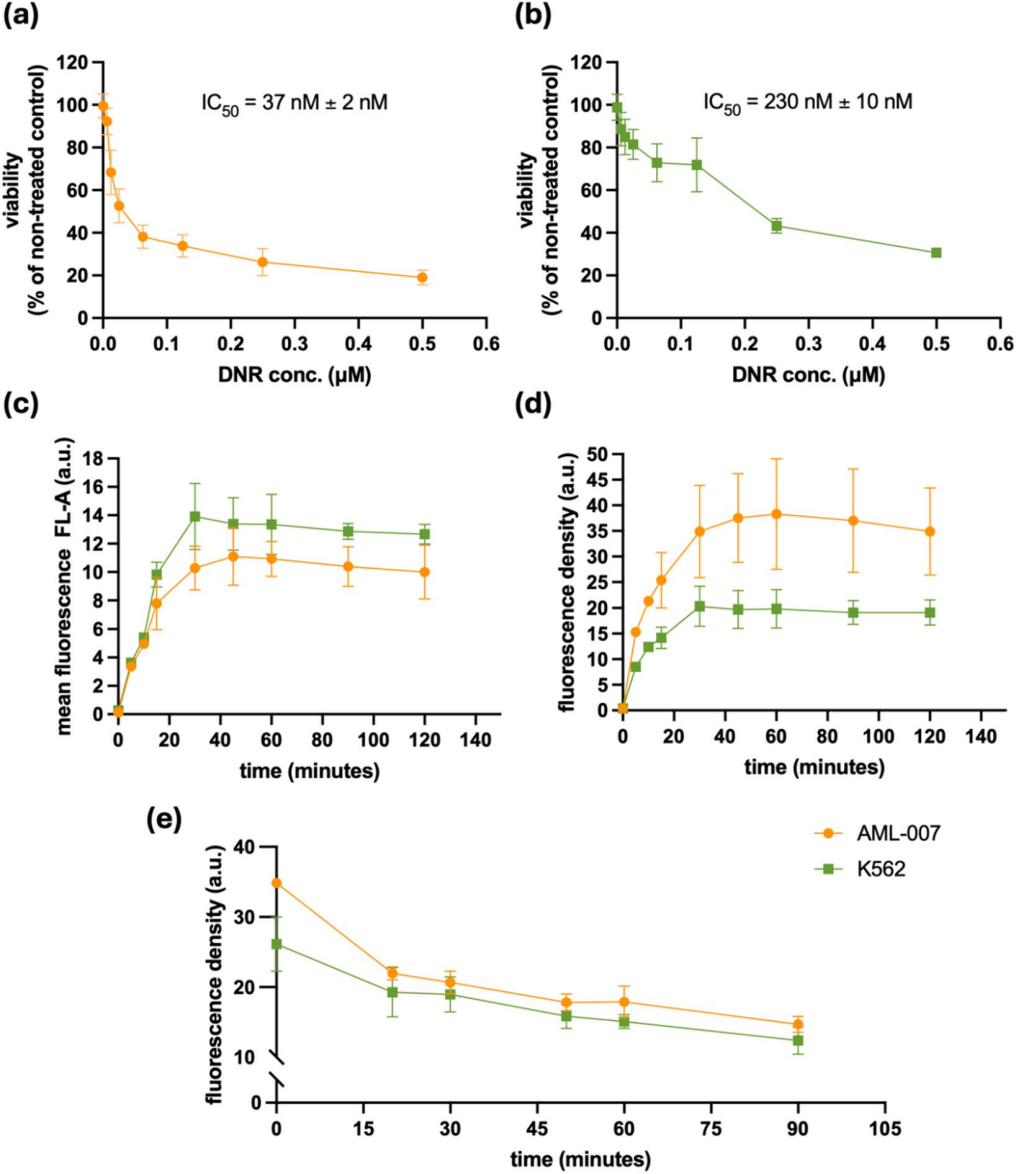
The viability of (**a**) AML-007 and (**b**) K562 measured in MTS assay after treatment with DNR. The untreated cells were set as 100% viability. Increase of DNR-related fluorescence accumulated from media with 5 µM DNR within the AML-007 and K562 cells over a time showed as an increase of measured (**c**) *FL-A*/10 000 or (**d**) fluorescence density (*FD*). (**e**) Decrease of *FD* of AML-007 and K562 after 30-minutes incubation with 5 µM DNR over the time. Averages along with standard deviation are shown on the graphs.

For the initial study, two cell lines with significantly different sensitivities to DNR were selected. To identify the appropriate model cell lines for the experiment, an MTS assay was performed. The K562 cell line, with an IC50 of 230 nM ± 10 nM (Fig. 1a), was chosen as the resistant model, while AML-007 cells, with an IC50 of 37 nM ± 2 nM (Fig. 1b), were chosen as the sensitive model.

To assess the quantity and kinetics of DNR accumulation within the cells, its fluorescent properties were utilized, and DNR was directly measured by cytofluorometry using a 575/26 nm filter. The cells were incubated with 5 µM DNR, washed, and DNR-related fluorescence (*FL-A*) was determined at various time points (Fig. 1c). A rapid increase in fluorescence was observed within the first 40 min of cell-drug exposure. However, longer incubations did not result in a further increase in DNR-related fluorescence.

Interestingly, no significant difference in *FL-A* was observed between the cell lines, with sensitive AML-007 cells showing slightly lower fluorescence readings. However, since *FL-A* reflects the overall fluorescence of the event (cell), which is also influenced by the size of the event, we normalized the DNR-related *FL-A* readout by dividing it by the *FSC-A* (forward scatter) value, which is proportional to the cell diameter. This corrected parameter, referred to as Fluorescence Density (*FD*), provides a more accurate measure independent of event size. Using *FD*, which can be considered analogous to the fluorescent drug concentration, we observed a significant difference in the kinetics of DNR accumulation between AML-007 and K562 cells (Fig. 1d). Both cell lines showed differences in the rate of drug accumulation and the maximum *FD* values. The maximum *FD* and the rate of increase were significantly lower in the resistant K562 cells compared to the sensitive AML-007 cells.

Given that drug catabolism and efflux are critical components of chemoresistance, we also studied the decay of DNR-related fluorescence density after an initial 30-minute incubation of cells with 5 µM DNR. DNR-derived fluorescence was assessed immediately after washing out the drug (time 0) and at subsequent time points (Fig. 1e). The decay of DNR *FD* was most rapid during the first 20 min, after which a gradual, slight decrease in *FD* was observed. Interestingly, beyond 20 min, there was no difference in *FD* kinetics or values between the low-IC_50_ AML-007 cells and the high-IC_50_ K562 cells. These results suggest that the most significant differences between AML-007 and K562 cells occur in the initial DNR influx, with less variation observed in drug efflux or catabolism.

### Brief exposure to DNR is sufficient for cytotoxicity induction

**Fig. 2:**
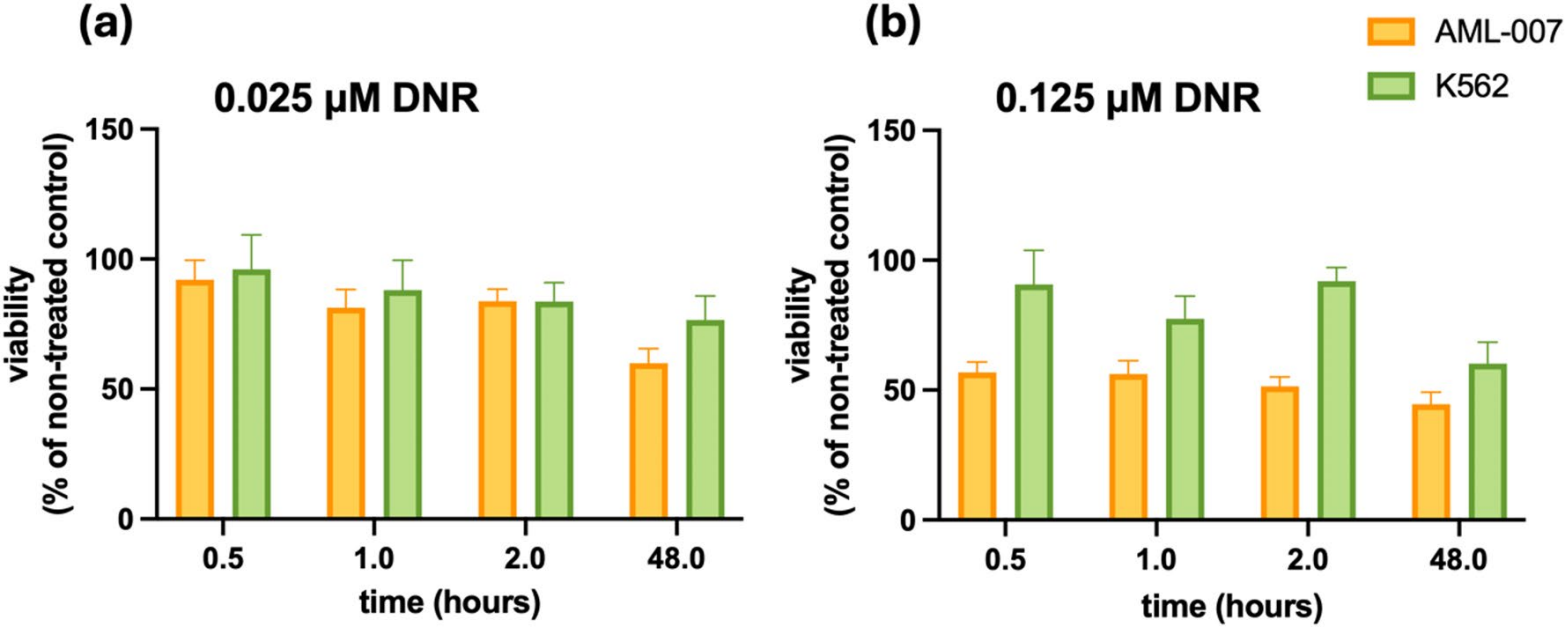
Viability of AML-007 and K562 cells measured after brief exposure to drug followed up 48 h incubation in fresh culture medium. The cells were treated with DNR over indicated time with (**a**) 0.025 µM or (**b**) 0.125 µM. The averages and standard deviations are shown on the graphs.

The collected data suggest that the initial influx of DNR may differentiate between resistant and sensitive cells. To further investigate this issue, we examined the cytotoxicity of DNR after brief exposures of 30, 60, or 120 min to DNR in concentrations of 25 nM and 125 nM (Fig. 2). At 25 nM, no differences in cell viability were observed between the two cell lines. However, at the higher concentration of 125 nM, a cytotoxic effect was observed in AML-007 cells, but not in K562 cells. Notably, even a 30-minute exposure was sufficient to differentiate between the DNR-susceptible AML-007 and the DNR-resistant K562 cells. These results highlight the biological relevance of short-term evaluations of susceptibility to DNR.

### The DNR concentration-dependent increase in cells fluorescence

**Fig. 3:**
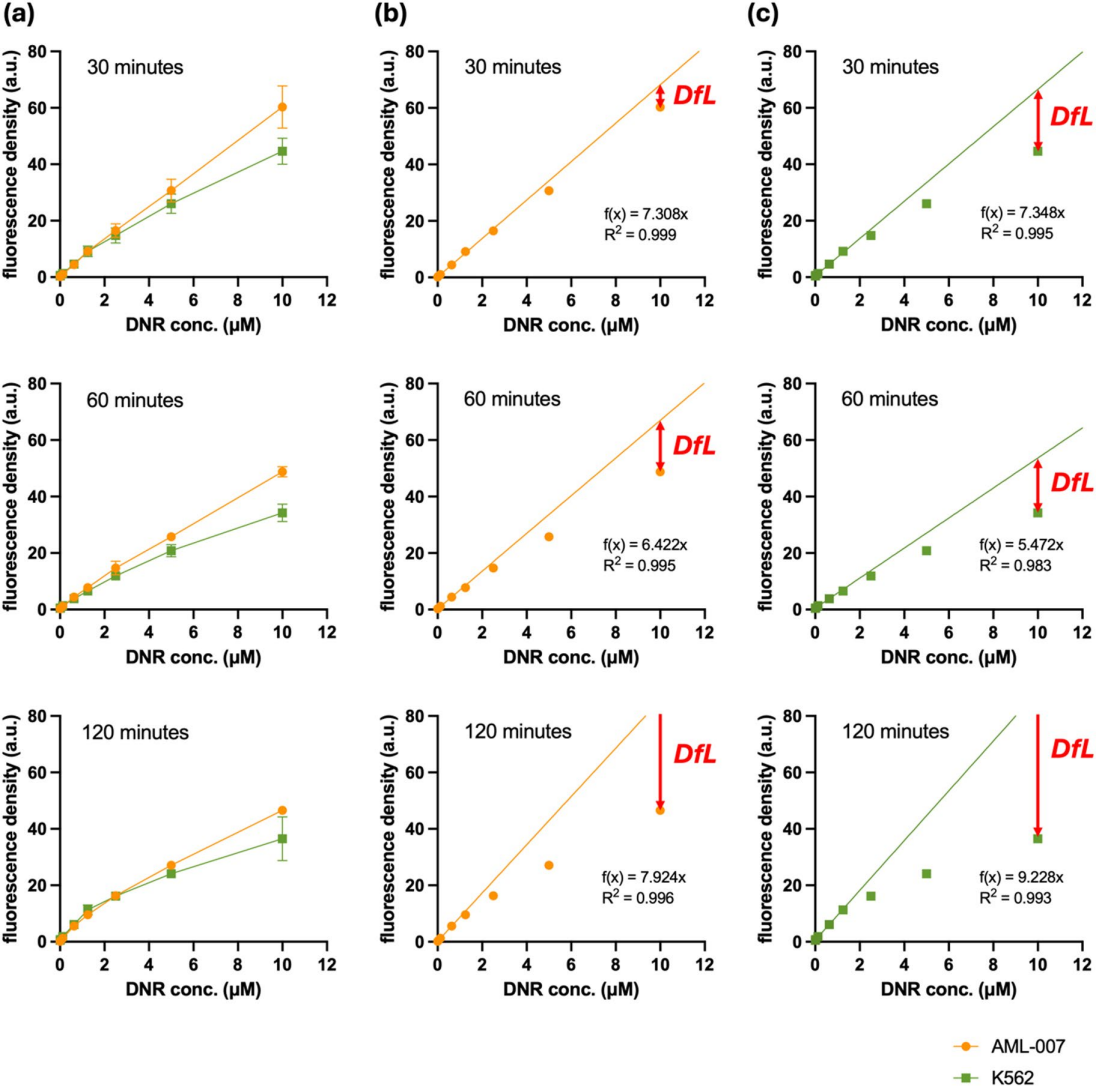
(**a**) Increase of DNR-related fluorescence reflecting accumulation of DNR within the AML-007 and K562 as a function of DNR concentration in treatment media during 30-, 60- or 120-minutes exposure. The averages and standard deviations are shown on the graphs. Increase of DNR-related fluorescence reflecting accumulation of DNR within the (**b**) AML-007 and (**c**) K562 as a function of DNR concentration in treatment media during 30-, 60- or 120-minutes exposure. Averages from 4 experiments are shown. The lines depict linear relationship observed at low concentrations. The Deviation from Linearity (*DfL*). Islets contain approximated formulas along with coefficient of determination R^2^.

In exploring the initial hours of drug-cell interactions, we investigated how sensitive and resistant cells acquire the drug depending on its concentration. Thus, the AML-007 and K562 cells were incubated in DNR with increasing concentrations ranging from 0.1 µM to 10 µM. As expected, fluorescence and *FD* increased with the DNR concentration in both cell lines (Fig. 3). Interestingly, there was only a slight difference in *FD* between the AML-007 and K562 cells in concentrations up to 1.25 µM. The difference between the cell lines became apparent only in samples incubated with DNR concentrations above 5 µM. A closer analysis of the concentration dependence revealed a nearly linear relationship between drug-related *FD* and DNR concentration at low concentrations, with the coefficient of determination (R²) for the linear approximation exceeding 0.98. However, when the DNR concentration reached 10 µM, a significant deviation from linearity was observed (marked as *DfL* in Fig. 3), especially in the K562 cells. Similar observations were made regardless of the drug exposure time. The deviation from linearity was more pronounced in K562 cells than in AML-007 cells, regardless of the time point used for evaluation.

### Multiparametric analysis of susceptibility to DNR

Two phenomena were identified that differentiate DNR-sensitive AML-007 cells from DNR-resistant K562 cells. The first is the lower *FD* value at higher drug concentrations, and the second is a significant deviation from the linear relationship between *FD* and DNR concentration at high drug concentrations. To describe and quantify these phenomena, we propose to exploit two parameters. The first is *FD*_*10*_, which is the *FD* measured after a 1-hour exposure to DNR in concentration of 10 µM. A high *FD*_*10*_ value indicates sensitivity to the drug. The second parameter, Deviation from Linearity (*DfL*), is defined as the difference between the expected *FD*_*10*_ value, calculated from the linear equation derived from low concentration data, and the measured *FD*_*10*_ value. A high *DfL* value indicates a significant deviation from the linear projection, suggesting that the cell’s drug clearance systems have become saturated or maximally activated. The rationale for using *DfL* is both technical and biological. Technically, *DfL* is calculated to capture the non-linear relationship observed between *FD* and increasing DNR concentration, providing a quantitative metric of this deviation. Biologically, the non-linearity (high *DfL*) is the functional signature of active, saturable drug clearance mechanisms (e.g., enhanced metabolism via NAD(P)H dehydrogenase, or efflux activity) in resistant cells (K562). At the diagnostic dose of 10 µM, these systems are maximally activated, effectively limiting the expected additional drug accumulation. In contrast, sensitive cells exhibit a more linear uptake, as their clearance capacity is lower. Therefore, *DfL* quantitatively captures the efficiency of the intrinsic cellular resistance machinery. The ability to maintain a higher concentration gradient suggests resistance. Since both phenomena can independently predict a high IC_50_, we combined them into a single parameter, termed the Susceptibility Index (*S-index*).

To assess whether these parameters could predict susceptibility to DNR (related to IC_50_), we tested additional non-adherent leukemic cell lines. The IC_50_ and cytofluorometric parameters (including *FSC-A*, *FL-A*, and their derivatives *FD*_*10*_, *DfL*, and *S-index*) were measured and calculated for these cell lines. Spearman’s rank correlation analysis (ρ) was then performed to evaluate the relationships between these parameters (Table 1). This approach revealed that IC_50_ does not correlate with *FSC-A*, raw measured fluorescence (*FL-A*), or even *FD*_*10*_, that differentiated K562 and AML-007 cells. Indeed, we observed a strong and significant correlation between IC_50_ and the *DfL* parameter (ρ = 0.806, *p* < 0.005). This correlation became even stronger for the *S-index* (ρ = 0.989, *p* < 0.001). Notably, we also observed an interdependence between *FSC-A* and *FL-A* fluorescence, which supports our proposal of *FD* as a parameter that accurately reflects the drug content within the cells, independent of cell size.

**Table 1:** Results of correlation analysis of Raw and calculated cytometry parameters with IC_50_ performed using data from panel of 11 lymphatic cell lines.

	IC_50_ (µM)	FSC-A	FL-A	FD_10_	DfL	S-index
IC_50_ (µM)	–	0.184	0.533	0.510	**0.806** *p* < 0.005	**0.989** *p* < 0.0001
FSC-A		–	**0.791** *p* < 0.005	0.109	0.105	0.164
FL-A			–	0.336	0.205	0.518
FD_10_				–	0.123	n.a
DfL					–	n.a
S-index						–

Statistically significant values are indicated in bold.

Spearman’s rank correlation coefficient ρ and *p* value are shown.

### High expression of the NADH dehydrogenase system players can contribute to resistance to daunorubicin of K562 cells

Next, we sought to investigate the reasons behind the high *DfL* observed in resistant cells. To address this, we performed a multistep bioinformatic analysis that focused on the proteome difference between Jurkat cells that exhibited an almost linear relationship between the DNR concentration and its accumulation within the cell and K562, which showed a high *DfL* (Table 2).

**Table 2:** Characteristic of K562 and Jurkat cells used in differential cluster identification bioinformatic analysis.

	IC_50_ (µM)	FSC-A	FL-A	FD_10_	DfL	S-index
K562	0.23	73 560	25 425	35	21	14
Jurkat	0.025	43 969	17 817	40	3	37

Both data types (proteomics and viability values) were available for 277 cell lines. We identified 752 proteins correlated with DNR viability values. Subsequently 150 out of 752 proteins displayed differential expression between Jurkat and K562. More specifically, we identified 55 proteins that were elevated in Jurkat cells compared to K562, and 95 proteins that were elevated in K562 cells compared to Jurkat. However, no statistically significant enrichment was found for the proteins elevated in Jurkat cells.

On the other hand, we found significant enrichment of proteins involved in NADH dehydrogenase activity and oxidoreductase activity that were elevated in K562 (Supplementary Materials, Table S1). Interestingly, most upregulated genes were assigned to clusters connected with NAD(P)H-coupled redox metabolism. It suggests that in K562 cells, enhanced DNR metabolism may be responsible for DNR clearance from the cells, which is reflected in their ability to handle high drug concentrations. This, in turn, leads to a higher *DfL* value and contributes to the observed resistance to DNR.

### Distribution of fluorescent drug and dyes within cells could differentiate sensitive and resistant cells

**Fig. 4:**
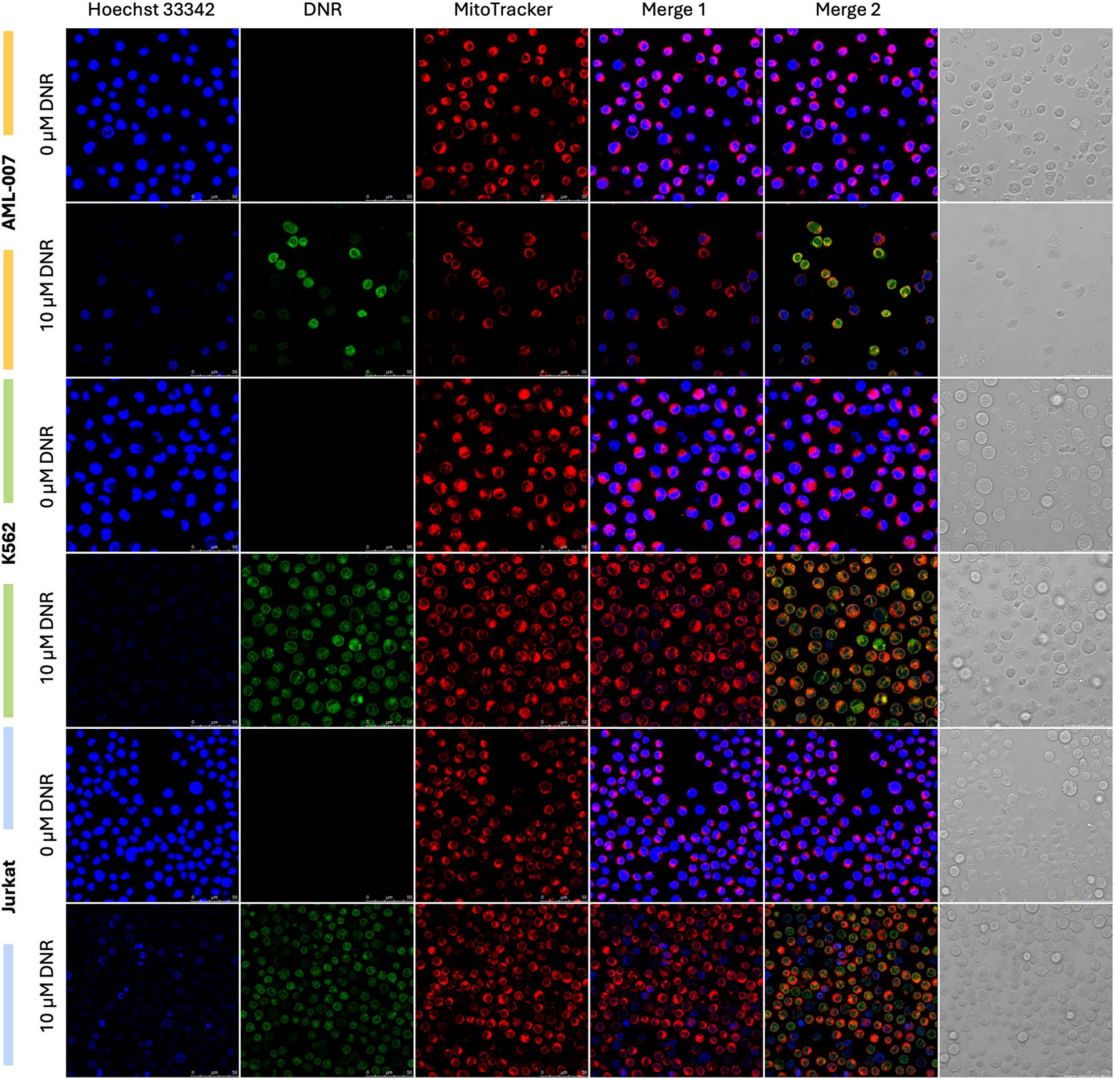
The confocal laser scanning microscopy images of the AML-007, K562 and Jurkat leukaemia cells after 2 h incubation with 10 µM DNR (green pseudocolor) and staining of cell nuclei with Hoechst 33,342 (blue pseudocolor) and mitochondria with MitoTracker (red pseudocolor). The channel superimposition with stained nuclei and mitochondria is shown in Merge 1, while stained nuclei, mitochondria and drug localization within the cell are shown in Merge 2. Transmission images are also shown. Scale bar in the lower right corner indicates 100 μm.

In order to visualize possible changes in cell morphology and determine the intracellular localization of fluorescent DNR in relation to selected cell structures, microscopic images of the leukemic lines selected for the experiment: AML-007, K562 and Jurkat, were recorded (Fig. 4).

Hoechst 33,342 emits blue fluorescence when bound to AT-rich sequences in the minor grove of double-stranded DNA^29^. In the control groups, all nuclei present in the sample were blue-stained. In the case of pre-incubating cells with DNR followed by the introduction of a nuclear dye, an interesting observation was made: not all nuclei were stained. For the DNR-sensitive cell lines (i.e., AML-007 and Jurkat), some nuclei were stained with the nuclear dye, while exhibiting negligible fluorescence from DNR. Conversely, cells with high DNR fluorescence lacked blue staining from the nuclear dye. In the case of the resistant K562 cell line, all cells displayed fluorescence from DNR, but no nuclei were visible with the nuclear dye, indicating an absence of nuclear staining. We hypothesize that this may be due to DNR’s ability to effectively insert its daunosamine fragment into the minor groove of DNA^30^, the nuclear dye target site.

Moreover, higher fluorescence intensity is observed within the sample with sensitive cells, which is consistent with the results presented above (including the proposed *DfL* parameter and its lower value, resulting from loading cells with the DNR drug). Resistant cells have lower fluorescence intensities (and therefore a higher *DfL* parameter – to compare with results above).

By interpreting the recorded microscopic images and in line with literature reports (Hajian et al., 2009), which state that DNR fluorescence can be quenched by its interaction with DNA, we observe that in sensitive cells, DNR binds to its target site, the appropriate region of DNA, leading to fluorescence quenching. In contrast, in resistant cells, mechanisms likely prevent DNR from binding to its target site, allowing the drug’s fluorescence to remain. This results in a distinct pattern of DNR distribution in sensitive cells (with both drug-loaded and drug-free cells), while resistant cells exhibit a more uniform pattern of drug fluorescence, indicating a homogeneity in drug uptake. It is also important to note that DNR significantly affects membrane permeability in sensitive cells^32^, which may lead to drug loss from the cells.

Therefore, considering the co-localization of the drug with intracellular structures, it appears feasible to visually distinguish sensitive cells from resistant ones using a short-term incubation with DNR, along with basic staining and imaging procedures.

### Leukemia cells response to daunorubicin treatment

**Fig. 5:**
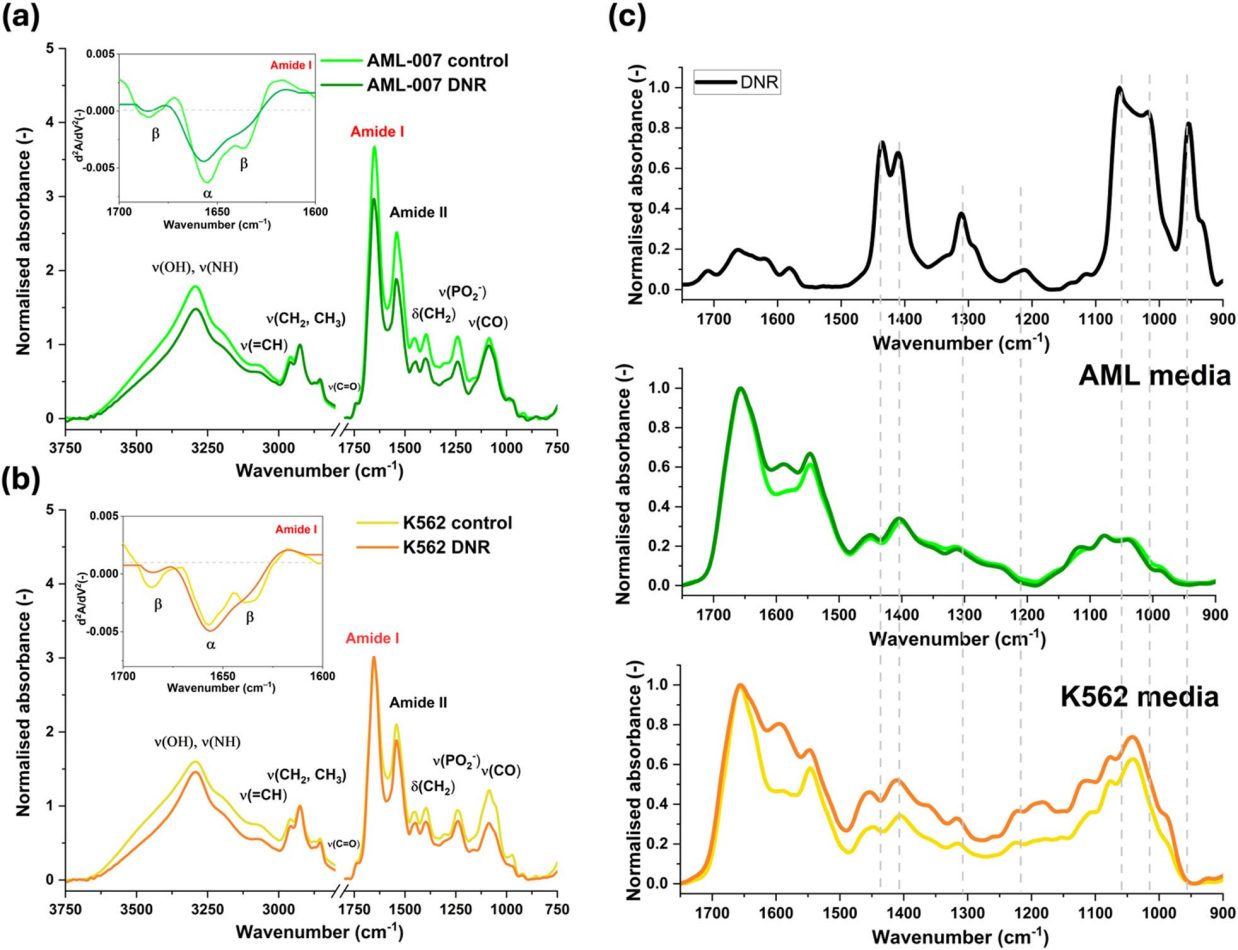
Averaged normalized spectra of (**a**) AML-007 and (**b**) K562 leukaemia cells for control and drug treatment (10 µM of daunorubicin, 2 h incubation time). The most important bands discussed below are marked on the FTIR spectra. 2nd derivative spectra in the Amide I range (1700 –1600 cm^− 1^) are shown and the band corresponding to α-helix and β-sheet structures are marked. (**c**) Normalized FTIR spectrum of DNR in DMSO (black line) in the 1750 –900 cm^− 1^. Normalized FTIR spectra of media used for cell culturing before and after DNR treatment.

Fourier-transform infrared spectroscopy (FTIR) was employed to analyze biochemical changes in leukemia cells following daunorubicin treatment. The key bands in the averaged spectra of AML-007 and K562 cells (Fig. 5) are listed in Table 3. Daunorubicin impacts membrane lipids, leading to noticeable spectral differences. A reduction in the intensity of bands at 2962 cm^−1^ and 2875 cm^−1^, which primarily correspond to the asymmetric and symmetric stretching of methyl groups, was observed in both leukemia cell types after treatment. The reduction in these band intensities suggests increased lipid packing and decreased membrane fluidity^33–35^ and it may also indicate inhibition of DNA methylation in drug-treated cells^36^.

**Table 3:** The tentative assignments of major bands in FTIR spectra of the studied leukemia cells^60^. Arrows indicate changes in the proper band intensity after DNR treatment.

AML-007	AML-007 DNR	K562	K562 DNR	Vibration	Assignment
Band position (cm^− 1^)					
3228	3286 ↓	3284	3286 ↓	νOH, νNH	Water, proteins (Amide A)
3063	3063 ↓	3063	3063 ↓	νNH	Proteins (Amide B)
3012	3012 ↓	3012	3012 ↓	ν(C = CH)	Unsaturated lipids
2962	2962 ↓	2962	2962 ↓	ν_asym_(CH_3_)	Lipids, proteins
2924	2924	2924	2924	ν_sym_(CH_2_)	Lipids, proteins
2875	2875 ↓	2875	2875 ↓	ν_asym_(CH_3_)	Lipids, proteins
2852	2852 ↑	2852	2852 ↓	ν_sym_(CH_2_)	Lipids, proteins
1741	1743 ↑	1741	1741 ↓	ν(CO)	Triglycerides and cholesterol esters
1684	1686 ↓	1684	1686 ↓	ν(CO), ν(CN)	Proteins (Amide I) : β-sheet
1655	1657 ↓	1655	1657 ↑	ν(CO), ν(CN)	Proteins (Amide I) : α-helix
1543	1545 ↓	1545	1545 ↓	δ(NH), ν(CN)	Proteins (Amide II)
1450	1446 ↓	1450	1446 ↓	δ(CH_2_)	Lipids, proteins
1394	1402 ↓	1394	1398 ↓	ν(COO^−^)	Lipids
1242	1238 ↓	1242	1238 ↓	ν_asym_(PO_2_^−^)	Nucleic acids, phospholipids, proteins
1086	1088 ↓	1086	1088 ↓	ν_sym_(PO_2_^−^)	Nucleic acids, phospholipids
1055	1057 ↓	1049	1055 ↓	ν(CO)	Carbohydrates, glycoproteins
966	966 ↓	968	966 ↓	ν(CN)	Nucleic acids

ν, stretching; δ, deformation.

The lipid phase transition is further supported by an increase in the 1460 cm^−1^ subband (CH₂ scissoring mode), which is particularly pronounced in K562 cells. In the FTIR spectra of AML-007 drug-treated cells, a shift of the 1740 cm band (ester bonds in phospholipid headgroups) to higher wavenumbers, accompanied by an increase in absorbance, was observed. This shift indicates structural changes in lipids due to oxidative stress, impacting membrane fluidity and transport functions^37^. A significant decrease in the intensity of this band was observed in K562 cells post-treatment, suggesting a reduction in unsaturated lipid content. This change may reflect lipid peroxidation or modifications in membrane fluidity induced by DNR.

Analysis of the amide bands region (1700 –1500 cm^−1^) provided insights into protein content and secondary structures. The intensity of Amide II band (N-H bending and C-N stretching in peptide bonds) at ca. 1543 cm^− 1^ decreased after DNR treatment, especially in the case of AML-007. The α/β ratio (1655/1684)^38^ generally increased, due to a significant lowering of β-sheet content. However, the intensity of the Amide I band at approximately 1655 cm^−1^ may be slightly influenced by C = C vibrations of cholesterol and other unsaturated lipid components, as well as by the presence of subbands at 1660 cm^−1^ and 1640 cm^−1^, which correspond to C–C ring vibrations of DNR^39^. These alterations in the Amide I and II bands can serve as indicators of apoptosis-induced structural changes in cells^40^.

The bands at 1242 and 1086 cm^− 1^ are assigned to nucleic acids and attributed to asymmetric and symmetric stretching of PO_2_ groups, respectively^41^. A decrease in the intensity of these bands in FTIR spectra of AML-007 cells after DNR treatment suggests significant changes in nucleic acid structure and integrity. Daunorubicin intercalates between DNA base pairs and leads to strand breaks and fragmentation. The decreasing of phosphate band intensities followed by a decrease in DNA absorbance at 966 cm^− 1^ (due to C-C/C-O stretching of deoxyribose-ribose vibration) is a hallmark of apoptosis in DNR-treated leukemia cells^42–44^.

The band at 1055 cm^−1^ in FTIR spectra can be attributed to glucose or carbohydrates, specifically to C–O, C–C, and C–OH stretching vibrations in saccharides^45^. The strong decrease of absorption in 1060–1050 cm^−1^ is indicative for K562 line.

In the FTIR spectrum of the cell medium (Fig. 5c) recorded after 2 h of treating K562 leukemic cells with daunorubicin, spectral line changes in the range of 1750 –900 cm^−1^ are observed, which can be attributed to the presence of the drug^39^ or its metabolite in the medium. A similar increase in the intensity of bands (ca. 1365, 1190, 1150 and 986 cm^− 1^) assigned to DNR is not observed in the case of the AML cell line medium.

## Discussion

Acute myeloid leukemia (AML) presents variable responses to anthracyclines like daunorubicin (DNR), with functional resistance mechanisms playing a significant role in treatment failure and relapse^1,4^. While genomic and cytogenetic markers are essential for classification and prognosis^3^, they do not fully account for chemoresistance, as metabolic and microenvironmental factors also contribute^2^. Several studies have highlighted the importance of functional assays in predicting chemotherapy responses^10,14^.

A critical methodological consideration in our study involved the quantification of intracellular drug levels. Conventional flow cytometry relies on Mean Fluorescence Intensity (MFI, or FL-A), but this parameter is inherently dependent on cell size (FSC-A). Given the natural morphological heterogeneity across our panel of leukemic cell lines, including primary AML samples, MFI was inadequate as it primarily reflected the total drug mass. To ensure a robust, size-independent comparison, we introduced Fluorescence Density (*FD*), defined as the ratio of FL-A to FSC-A. This crucial normalization step allows *FD* to serve as an accurate proxy for the internal drug concentration, validating its use as a foundational metric for our diagnostic assay. Next, we propose a flow cytometry-based functional assay to assess daunorubicin accumulation and efflux, using its intrinsic fluorescence to generate two novel parameters – Susceptibility Index (*S-index*) and Deviation from Linearity (*DfL*). These parameters provide a real-time, quantitative measure of drug resistance, correlating strongly with IC_50_ values, which makes them a promising tool for predicting treatment response.

Flow cytometry showed that *S-index* and *DfL* accurately distinguish sensitive and resistant cells. The *S-index* quantifies intracellular daunorubicin retention, with higher values indicating greater drug accumulation and sensitivity. *DfL* represents deviations from expected drug uptake patterns at increasing concentrations. The *S-index* quantifies intracellular daunorubicin retention, with higher values. Resistant K562 cells displayed a high *DfL*, reflecting their ability to limit additional drug accumulation at high doses. In contrast, sensitive AML-007 cells showed sustained drug uptake and a lower *DfL*. These fluorescence-based parameters offer a clinically relevant alternative to traditional long-term cytotoxicity assays, avoiding variability associated with dye-based efflux markers such as rhodamine or calcein-AM^8,9^. Prior research has demonstrated that P-glycoprotein (MDR1) expression plays a key role in efflux-mediated resistance in leukemia, thereby reinforcing the rationale for using flow cytometry-based methods to assess drug resistance^46,47^. Flow cytometry also holds potential for identifying new, highly potent cytotoxic anthracyclines^48^.

Given the importance of accurately reflecting the clinical context of AML treatment, it is essential to consider that daunorubicin is typically administered in combination with cytarabine in the standard 7 + 3 induction regimen. In this study, we focused on daunorubicin because it represents a primary indicator of resistance to the broader class of anthracyclines. The presented assay is designed to provide a rapid, one-hour functional assessment of resistance, a parameter that cannot be evaluated using long-term combination protocols. Although cytarabine is not naturally fluorescent, the assay serves as a diagnostic platform that can be expanded to include other anthracyclines or fluorescently traceable drugs and potentially adapted for combination studies once suitable labeling and measurement strategies are established. Future work could extend this approach to combination treatments, particularly daunorubicin and cytarabine, to better reflect clinical practice and to explore multidrug resistance mechanisms^5–7^.

Confocal microscopy revealed that daunorubicin was predominantly localized in the nucleus of AML-007 cells, while it remained primarily in cytoplasm of K562 cells. This suggests an active resistance mechanism that prevents nuclear drug accumulation, thereby reducing its cytotoxic activity. These findings align with previous studies on anthracycline resistance, where P-glycoprotein-mediated drug efflux and membrane sequestration were identified as key contributors^8,49^.

Furthermore, FTIR spectroscopy further confirmed biochemical differences in membrane composition between the two cell lines. K562 cells exhibited oxidative stress-related lipid modifications, leading to membrane rigidification and possibly enhanced drug efflux. This was reflected by spectral shifts in lipid-associated absorption bands. These changes likely contribute to reduced intracellular drug retention and altered transport dynamics, reinforcing the role of membrane remodeling in daunorubicin resistance^33,34^. Previous studies have demonstrated that membrane fluidity alterations influence drug transport in multidrug-resistant leukemia cells, supporting our findings^11,24^. Recent studies highlight the complex mechanisms underlying chemoresistance in cancer, particularly focusing on membrane transport proteins and metabolic adaptations. Membrane transporters play a crucial role in drug efficacy and toxicity by regulating cellular influx and efflux, with their altered expression contributing to chemoresistance^25^. Additionally, Fourier-transform infrared spectroscopy has been used to study lipid reorganization in leukemia models, further validating our approach^37,50^.

Beyond membrane-associated resistance, metabolic detoxification also plays a crucial role. Bioinformatic analysis revealed upregulation of NAD(P)H-dependent oxidoreductases in K562 cells, consistent with enhanced metabolic clearance of daunorubicin. Anthracyclines undergo enzymatic inactivation through NADH dehydrogenases, and K562’s increased expression of these enzymes suggests faster drug metabolism, reducing its intracellular concentration and effectiveness^25,51^. This metabolic adaptation, in combination with drug efflux mechanisms, likely accounts for K562’s ability to withstand high drug doses despite prolonged exposure^4^. These findings are further supported by recent studies showing that cancer cells can reprogram metabolic pathways to resist chemotherapeutic stress, particularly through altered glycolysis and mitochondrial function^52^. The observed increase in NADH dehydrogenase expression aligns with broader metabolic shifts that enable resistance across multiple cancer types.

## Future directions and ferroptosis resistance

While our study focuses on daunorubicin resistance through drug efflux and metabolic detoxification, recent evidence suggests that the regulation of oxidative stress also plays a crucial role in drug resistance. Ferroptosis, a non-apoptotic form of cell death driven by lipid peroxidation, has been identified as a potential vulnerability in cancer cells. However, several studies indicate that leukemia cells can develop resistance to ferroptosis through antioxidant defence mechanisms^53,54^. Given that membrane rigidity and lipid remodelling were observed in our FTIR results, it is possible that ferroptosis-related pathways contribute to daunorubicin resistance. Future research should investigate whether targeting ferroptosis regulators, such as GPX4 and NFE2L2 (NRF2), could enhance the sensitivity of resistant leukemia cells to daunorubicin.

Taken together, these results provide a comprehensive understanding of daunorubicin resistance: K562 cells limit drug uptake, prevent nuclear entry, and rapidly metabolize daunorubicin, while AML-007 cells accumulate the drug intracellularly, leading to greater sensitivity. By integrating flow cytometry, FTIR spectroscopy, confocal microscopy, and bioinformatics, this study establishes a robust framework for assessing chemoresistance. The strong correlation between the *S-index* and *DfL* with IC_50_ values suggests that these parameters could serve as functional biomarkers for daunorubicin susceptibility, with potential applications in clinical decision-making (Blom et al., 2017; Meijer et al., 2017).

If validated in patient-derived AML samples, this assay could enable real-time, personalized drug resistance profiling, facilitating tailored treatment strategies to improve patient outcomes^21^. Furthermore, future research should explore whether the *S-index* and *DfL* are predictive for other chemotherapy agents, such as cytarabine or FLT3 inhibitors^16,17^. Further studies should also incorporate metabolic inhibitors or gene knockdown experiments to confirm the direct role of NADH dehydrogenases in daunorubicin detoxification^25^. Addressing these avenues will help develop a clinically viable, rapid functional test for predicting chemotherapy response in AML.

## Conclusions

Short exposure to DNR is sufficient to produce different outcomes in sensitive and resistant cells. The flow cytometric parameters (*DfL*, *S-index*) derived from a brief functional assay are highly correlated with IC_50_ and may discriminate cell lines that are sensitive and resistant to DNR. The resistance to DNR can be facilitated by the high expression of NAD(P)H-coupled redox system players.

## Methods

### Cell culture conditions and materials

AML-007, AML-009, AML-017, AML-017 A, and AML-017B are non-commercial primary leukemia cell lines derived from the bone marrow of patients diagnosed with AML at the Clinic of Hematology, Blood Neoplasms, and Bone Marrow Transplantation, Wrocław Medical University (Bioethics Committee approval no. 593/2012). Primary cell lines were cultured in Advanced RPMI 1640 medium (Gibco, Thermo Fisher Scientific, Waltham, MA, USA). Jurkat, H3d, and B10 cells were cultured in RPMI 1640 medium (Ludwik Hirszfeld Institute of Immunology and Experimental Therapy, Polish Academy of Sciences, Wrocław, Poland), while U937, HL-60, and K562 cells were maintained in high-glucose RPMI 1640 medium (same institution). All culture media were supplemented with 10% fetal calf serum (FCS; Gibco, Thermo Fisher Scientific, Waltham, MA, USA).

### Toxicity studies

Cells were seeded in 96-well plates (Corning Life Sciences, Kennebunk, ME, USA) and treated with daunorubicin (DNR; Alfa Aesar, Ward Hill, MA, USA) at concentrations of 0.0063, 0.0125, 0.025, 0.063, 0.125, 0.25, and 0.5 µM. Following 48 h of incubation at 37 °C in a humidified atmosphere containing 5% CO₂, cell viability was assessed using the MTS assay. Specifically, 20 µl of CellTiter 96^®^ AQueous One Solution Reagent (Promega, Madison, WI, USA) was added to each well. After a 2-hour incubation, absorbance at 490 nm was measured using a Wallac 1420 Victor 2 plate reader (PerkinElmer, Waltham, MA, USA).To evaluate cell viability following short-term exposure to DNR, K562 and AML-007 cells were treated with 0.025 or 0.125 µM DNR for 30 min, 1 h, or 2 h. After the indicated incubation periods, cells were washed three times with phosphate-buffered saline (PBS; Ludwik Hirszfeld Institute of Immunology and Experimental Therapy, PAS, Wrocław, Poland) and reseeded into 96-well plates in fresh culture medium. Cells were then incubated for 48 h at 37 °C in a humidified 5% CO₂ atmosphere. Subsequently, viability was determined using the MTS assay, with 20 µl of CellTiter 96^®^ AQueous One Solution Reagent added per well. After 2 h, absorbance at 490 nm was again measured using the Wallac 1420 Victor 2 plate reader (PerkinElmer, Waltham, MA, USA).

### Assessment of daunorubicin influx and efflux

Daunorubicin influx was assessed by incubating cells with 5 µM DNR at 37 °C for 5, 10, 15, 30, 45, 60, 90, and 120 min, starting with the longest time points. For concentration-dependent analyses, cells were exposed to 0.16, 0.33, 0.67, 1.25, 2.5, 5, or 10 µM DNR for 30, 60, or 120 min. Following incubation, cells were washed twice with phosphate-buffered saline (PBS) and resuspended. Intracellular DNR levels were quantified by measuring the mean fluorescence intensity (488 nm excitation/575 nm emission) using an LSRFortessa™ flow cytometer (Becton Dickinson, Franklin Lakes, NJ, USA).

Efflux studies were performed by incubating cells with 5 µM DNR at 37 °C for 30 min, followed by a PBS wash and resuspension in fresh culture medium. Cells were then maintained at 37 °C for 20, 30, 50, 60, or 90 min. After each time point, cells were washed three times with PBS and resuspended in cold buffer. The residual intracellular DNR content was determined via flow cytometry. Data analysis was conducted using Flowing Software version 2.5.1 (University of Turku, Turku, Finland).

### Spectroscopic characterization of the biochemical composition of leukemia cells

Fourier-transform infrared spectroscopy (FTIR) spectra of AML-007 and K562 leukemia cells after 2-hour incubation with 10 µM daunorubicin (DNR, shown in green) were recorded using an iN10 FTIR microscope (Thermo Scientific, USA) equipped with a 15× objective and a cadmium-mercury-telluride (MCT-A) detector cooled with liquid nitrogen. The microscope was continuously purged with dry air to minimize atmospheric interference. As references, spectra of all culture media (before and after incubation), daunorubicin in DMSO, and the DMSO solvent were also collected.

Samples were applied onto BaF₂ windows and allowed to dry at room temperature. Spectra were acquired in transmission mode across a wavenumber range of 4000–675 cm^−1^, using 128 scans per sample (512 scans for background) at a resolution of 4 cm^−1^. The aperture size was set to 50 × 50 μm. Each measurement was repeated 3–4 times to ensure reproducibility. Automatic atmospheric correction was performed using OMNIC software (version 8, Thermo Fisher Scientific, USA), and further data preprocessing and analysis were carried out with OriginPro 2023b SR1 (OriginLab Corporation, USA). Preprocessing steps included baseline correction, smoothing with a Savitzky–Golay filter (polynomial order 2, window size 15), and normalization to the absorbance of the ν_asCH₂ band (2924 cm^−1^).

Principal Component Analysis (PCA) was applied to the fingerprint region (1800–750 cm^−1^) for preliminary data reduction prior to methylation level analysis.

### Parameters calculations and statistics

The Fluorescent Density (*FD*) was calculated by dividing mean *FL-A* value (LP550, 575/26 nm filter) by mean *FSC-A* value of cell population excluding multiplets and multiplying the result by 100. The data from the first four concentration points (DNR ≤ 1.25 µM) were used for linear approximation, with the intersection fixed at (0, 0). This formula was then applied to predict the *FD* at 10 µM. The difference between the predicted and measured *FD* values defines the variation from linearity (*DfL*) parameter, which was rounded to the nearest value. This formula serves as the definition of the susceptibility index.

For correlation analysis, Spearman’s rank correlation coefficient (ρ) was calculated and is presented in the tables along with the corresponding p-values.

### Bioinformatic studies and statistical analyses

Data on log fold-change viability values of 564 cell lines to DNR was downloaded from DepMap portal (PRISM Repurposing 19Q3 Primary Screen)^55,56^. Proteomic data for 375 cell lines, including Jurkat and K562, published by Nusinow et al. was downloaded from DepMap portal^57^. Both data types were available for a total 277 cell lines.

All statistical analyses were performed in the R environment. For the proteomic data, proteins with more than 10% missing values were removed. The remaining missing values were imputed using the *impute* R package with nearest-neighbour averaging, enabling the analysis of 8000 proteins^58^.

The correlation between DNR viability values and protein expression levels was calculated using Spearman’s correlation. Proteins with an absolute correlation ≥ 0.15 and an adjusted *p*-value ≤ 0.05 were retained for downstream analyses. To select genes differentially expressed in Jurkat compared to K562, a threshold of absolute difference ≥ 1 was applied. Enriched pathways were assessed using the WebGestalt portal through over-representation analysis (ORA) and gene ontology – molecular function, with a significance threshold of adjusted *p*-value ≤ 0.05^59^.

### Visualization of cell morphology and DNR co-localization with cellular structures

Cells from selected leukemia lines (AML-007, K562, and Jurkat) were incubated for 2 h under standard conditions (37 °C, 5% CO2) in complete medium supplemented with 10 µM DNR. After incubation, cells were centrifuged (180 × g, 7 min), washed in PBS (Invitrogen, Gibco, Thermo Fisher Scientific, Waltham, MA, USA), and resuspended in the staining solution. The staining solution contained 2 drops of NucBlue™ Live ReadyProbes™ Reagent (Hoechst 33342, Invitrogen, Thermo Fisher Scientific, Waltham, MA, USA) per 1 ml of solution and MitoTracker™ Deep Red FM (Invitrogen, Thermo Fisher Scientific, Waltham, MA, USA) at a final concentration of 300 nM in PBS. Cells were incubated for 45 min in the dark under standard conditions. Afterward, the cells were centrifuged, washed twice in PBS, and resuspended in the appropriate complete medium for each cell line.

Images were acquired using a Leica TCS SPE confocal microscope (Leica Microsystems, Wetzlar, Germany) equipped with diode excitation lasers at wavelengths of 405 nm for Hoechst 33,342 (nuclei represented in blue pseudocolor), 488 nm for DNR (drug intracellular accumulation represented in green pseudocolor), and 635 nm for MitoTracker™ (mitochondria represented in red pseudocolor). Image acquisition was performed using LAS AF software (Leica Microsystems, Wetzlar, Germany).

## Supplementary Information

Below is the link to the electronic supplementary material.

Supplementary Material 1

## Data Availability

The data that support the findings of this study are available from the corresponding author upon reasonable request. The datasets analyzed during the current study are available in the Cancer Dependency Map portal depmap.org repositories, dois: 10.1016/j.cell.2019.12.023, 10.1038/s43018-019-0018-6.

## Abbreviations

AML: Acute myeloid leukemia
DfL: Deviation from linearity
DNR: Daunorubicin
FCS: Fetal calf serum
FL: A–fluorescence area
FSC: A–forward scatter area
FD: Fluorescence density
FD10: Fluorescence density at 10 µM
FTIR: Fourier–transform infrared spectroscopy
MDR: Multidrug resistance
S-index: Susceptibility index
