# Supplementary material for: Flow cytometry-based targeted diagnostics for rapid assessment of daunorubicin resistance in acute myeloid leukemia
Source: Sci Rep. 2025 Dec 9;16:1214. doi: 10.1038/s41598-025-30844-2 (PMC12789628; doi:10.1038/s41598-025-30844-2)
Supplement: Supplementary file 1 — Supplementary Material 1 [file 41598_2025_30844_MOESM1_ESM.pdf]

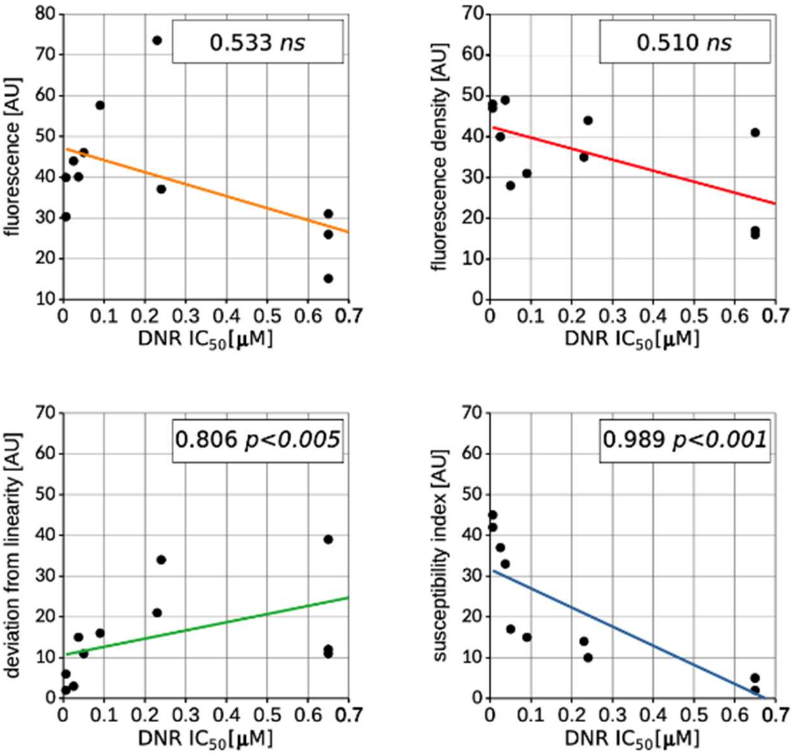

Figure S1 Correlation graphs of *FL-A*, *FD*, *DfL* and *S-index* parameters with DNR IC_{50} in 11 lymphatic cell lines (i.e., AML-007, AML-009, AML-017, AML-017A, AML-017B, Jurkat, H3d, B10, U937, HL-60, K562). Spearman's rank correlation coefficient ρ and p value are provided. *ns* – nonsignificant.

Table S1 Expression enrichments over Jurkat cells observed in K562 cells revealed via differential Cluster Identification bioinformatic analysis.

Cluster identification	Ratio	*p-value*	FDR
NADH dyhydrogenase (ubiquinone) activity GO:008137	40.764	$8.1157 \cdot 10^{-13}$	$6.2565 \cdot 10^{-10}$
NADH dehydrogenase (quinone) activity GO:0050136	40.764	$8.1157 \cdot 10^{-13}$	$6.2565 \cdot 10^{-10}$
NADH dehydrogenase activity GO:0003954	39.897	$9.9998 \cdot 10^{-13}$	$6.2565 \cdot 10^{-10}$
oxidoreductase activity, acting on NAD(P)H, quinone or similar compound as acceptor GO:0016655	31.253	$1.0320 \cdot 10^{-11}$	$4.8425 \cdot 10^{-9}$
oxidoreductase activity, acting on NAD(P)H GO:0016651	17.690	$1.9205 \cdot 10^{-9}$	$7.2097 \cdot 10^{-7}$
oxidoreductase activity GO:0016491	4.2753	$1.7207 \cdot 10^{-6}$	$5.3828 \cdot 10^{-4}$
RNA binding GO:0003723	2.7295	$1.4928 \cdot 10^{-5}$	$4.0027 \cdot 10^{-3}$
organophosphate ester transmembrane transporter activity GO:0015605	23.15	$2.8659 \cdot 10^{-4}$	$6.3257 \cdot 10^{-2}$
ADP transmembrane transporter activity GO:0015217	69.45	$3.3701 \cdot 10^{-4}$	$6.3257 \cdot 10^{-2}$
purine nucleobase transmembrane transporter activity GO:0005345	69.45	$3.3701 \cdot 10^{-4}$	$6.3257 \cdot 10^{-2}$
